# Chirality-sorted carbon nanotube films as high capacity electrode materials†

**DOI:** 10.1039/c8ra03963a

**Published:** 2018-08-30

**Authors:** Katarzyna Krukiewicz, Maciej Krzywiecki, Manus J. P. Biggs, Dawid Janas

**Affiliations:** Department of Organic Chemistry, Bioorganic Chemistry and Biotechnology, Silesian University of Technology B. Krzywoustego 4 44-100 Gliwice Poland dawid.janas@polsl.pl +48 32 2371082; CÚRAM – Centre for Research in Medical Devices, National University of Ireland 118 Corrib Village Galway Ireland; Department of Physical Chemistry and Technology of Polymers, Silesian University of Technology M. Strzody 9 44-100 Gliwice Poland; Institute of Physics – CSE, Silesian University of Technology Konarskiego 22B 44-100 Gliwice Poland

## Abstract

Carbon nanomaterials show great promise for a wide range of applications due to their excellent physicochemical and electrical properties. Since their discovery, the state-of-the-art has expanded the scope of their application from scientific curiosity to impactful solutions. Due to their tunability, carbon nanomaterials can be processed into a wide range of formulations and significant scope exists to couple carbon structures to electronic and electrochemical applications. In this paper, the electrochemical performance of various types of CNT films, which differ by the number of walls, diameter, chirality and surface chemistry is presented. Especially, chirality-sorted (6,5)- and (7,6)-based CNT films are shown to possess a high charge storage capacity (up to 621.91 mC cm^−2^), areal capacitance (262 mF cm^−2^), significantly increased effective surface area and advantageous charge/discharge characteristics without addition of any external species, and outperform many other high capacity materials reported in the literature. The results suggest that the control over the CNT structure can lead to the manufacture of macroscopic CNT devices precisely tailored for a wide range of applications, with the focus on energy storage devices and supercapacitors. The sorted CNT macroassemblies show great potential for energy storage technologies to come from R&D laboratories into real life.

## Abstract 2

Films from carbon nanotubes show great promise for energy storage devices.

## Graphical abstract

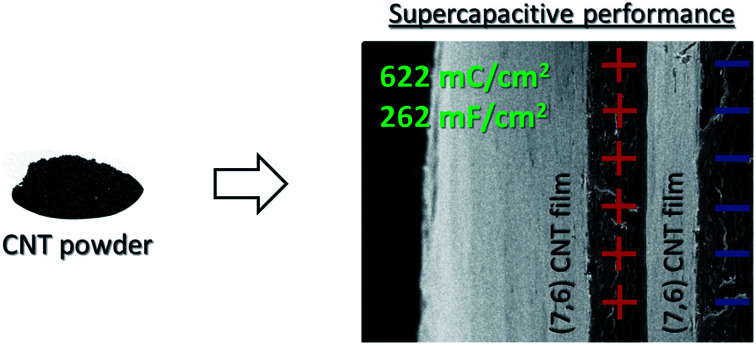

## Introduction

Carbon nanomaterials such as carbon nanotubes (CNTs) or graphene have shown great promise for a wide range of applications due to their excellent electrical,^1–3^ mechanical,^4–6^ thermal^7–9^ and optical properties.^10–13^ Since their discovery,^14,15^ the state-of-the-art has matured enough to prove the concept of many nanocarbon-based devices^2,16–19^ and to provide practical implementation. For instance, carbon nanomaterials have been employed successfully as atomic force microscopy probes,^20^ in the manufacture of light-weight bicycle frames^21^ and as light absorbing materials.^22^ Carbon nanomaterials can exist in numerous forms, differing in structure, size, chemistry, physicochemical and mechanical properties^23^ and it is possible to further enhance the desired properties of these materials through chemical and physical functionalization.^24–26^ Critically, significant leverage exists still for improving the efficacy of carbon nanomaterials in specific applications through tailoring the chemistry, and structure of carbon material formulation at the micro and nanoscales.

As the global energy demand increases at an exponential rate, there is a growing interest in the elaboration of new materials to improve energy management and carbon nanomaterials have been considered as a possible solution to this problem. They have been envisioned in next-generation wiring approaches (light weight, durability, high performance, environmentally friendly synthesis, *etc.*),^27^ employed in electrical machines^28^ for energy transportation. Moreover, they also offer beneficial properties for energy storage as vital parts of batteries,^29–32^ supercapacitors^33–35^ or hydrogen storage materials^36–38^ as described by Dai *et al.*^39^ In particular, the approach to use carbon nanomaterials as electrochemical capacitors has attracted significant R&D attention, mainly because of their high power storage capacity and long life cycle relative to traditional battery materials. For such application to be successful, carbon nanomaterials must have a high surface area and appropriate electrochemical performance in terms of capacitance governed by either faradaic or non-faradaic processes, depending on the mechanism of charge transfer between the electrode and the electrolytic solution. CNTs and graphene fulfil these criteria and offer competitive advantages owing to their stable molecular structure and 2D formulations; pure carbon cloth has the areal capacitance of 2 mF cm^−2^ (ref. 40) pure CNT electrode was found to possess the areal capacitance of 50 mF cm^−2^,^41^ and free-standing flexible hybrid papers made up of porous carbon particles combined with graphene sheets exhibited 103 mF cm^−2^.^42^ The addition of CNTs or graphene has been also reported to significantly enhance the performance of supercapacitor materials through the increase in the rate of the non-faradaic process, as it was observed for SnO_2_/CNT (capacitance of 4.42 mF cm^−2^),^43^ graphene/MnO_2_ (12.4 mF cm^−2^),^44^ activated carbon/PVDF/graphite/MWCNT composites (90.7 mF cm^−2^),^45^ graphene hydrogel (402 mF cm^−2^)^46^ and polypyrrole/CNT (1 F cm^−2^).^47^ Furthermore, carbon-derived nanostructures can help to overcome the problem of low areal specific capacitance that limits the application of solid-state supercapacitors in ultrathin film electrodes.^46^

In this report, we present on free-standing CNT films made of various types of CNTs, which differ by the number of walls, tube diameter, chirality and surface chemistry and show that these materials possess advantageous electrochemical properties, including exceptionally high capacitance, large effective surface area and beneficial charge/discharge characteristics without addition of any external species or supporting matrices. In particular, single-wall chirality-defined (6,5) and (7,6) CNTs revealed excellent charge-storage capacity (CSC) performance with measured CSC one order of magnitude higher than that of a platinum reference and an effective surface area enlargement factor of more than 10. It was confirmed that by careful synthesis of CNTs it is possible to reduce the wall defect frequency and obtain highly conductive films with significantly reduced impedance relative to commercially available CNTs and charge/discharge characteristics suitable for supercapacitor applications.

## Experimental

### Materials

Four free standing CNT material formulations were employed in this study: industrial-grade commercial multi-wall CNTs NC7000 (Nanocyl) as well as chirality-enriched (7,6) and (6,5) CNTs (SouthWestNanoTechnologies). In addition, we studied multi-wall CNT carpets formulated by chemical vapor deposition (CVD) of toluene and ferrocene according to a procedure reported previously.^48^

### Preparation of CNT films

The aforementioned materials were used for the preparation of free-standing CNT films of predetermined structure according to a method developed previously.^49^ In brief, described CNT formulations were sonicated in iso-propanol in the presence of a binding agent (ethyl cellulose) until a uniform dispersion was obtained. Then, the material was deposited onto a Kapton foil by spray coating, detached from the substrate and separated from the binder by flash annealing. Specimens of 28.5 mm^2^ area were used for the study.

### Characterization

Scanning Electron Microscopy and Transmission Electron Microscopy (SEM – FEI Nova NanoSEM at 10 kV accelerating voltage, TEM – Tecnai Osiris FEGTEM at 200 kV accelerating voltage) were used to visualize the microstructure of the materials.

Raman spectroscopy (Renishaw Ramascope 1000 with 633 nm emission wavelength) was used to analyze the surface chemistry. A ratio of intensity of disorder peak (D) to the intensity of peak of vibrations of graphitic lattice (G) gave information about the pristinity of the samples.

XPS investigations were carried out with PREVAC EA15 hemispherical electron energy analyzer equipped with 2D-MCP detector. The samples were irradiated with X-ray source (PREVAC dual-anode XR-40B, Al-Kα line, energy 1486.60 eV). The system base pressure was 2 × 10^−8^ Pa. For the survey spectra the scanning step was set to 0.9 eV with pass energy 200 eV while for particular energy regions to 0.05 eV with pass energy 100 eV. All of the measurements were performed with analyzer's axis perpendicular to samples' plane. The binding energy (BE) scale of the analyzer was calibrated to Au 4f_7/2_ (84.0 eV). Recorded data were fitted utilizing CASA XPS® embedded algorithms and relative sensitivity factors. Shirley function was used for the background subtraction. The estimated uncertainty for components' energy position determination was 0.1 eV.

Thermogravimetric analysis (TGA – Mettler Toledo TGA/DSC system) was used to measure changes in chemical and physical properties as a function of temperature. The samples were heated from room temperature to 1000 °C at 10 °C min^−1^ in the flow of air (20 ml min^−1^). The gas adsorption parameters were measured using nitrogen adsorption at 77 K (Tristar3000). 100 mg samples were used for analysis. BET model was employed to calculate specific surface area.

### Electrochemical evaluation

The voltammetric experiments were carried out using PARSTAT 2273 Advanced Electrochemical System (Princeton Applied Research) in a three-electrode electrochemical Teflon cell equipped with O-ring, CNT working electrode, Ag/AgCl (3 M KCl) reference electrode and platinum coil counter electrode. CV scans were recorded in 0.1 M KCl solution, in the potential range from −1.0 V to 1.2 V at a scan rate of 0.1 V s^−1^. CV curves were used to determine charge storage capacity (CSC), calculated as the electric charge integrated under corresponding CV curve during one CV cycle.^50^ To ensure the good infiltration of electrolyte into CNT films, the samples were immersed in the solution of electrolyte for one hour before the electrochemical measurements. The relative contribution from faradaic and diffusion controlled processes was investigated by conducting CV experiments at different voltage scan rates, from 10 mV s^−1^ up to 200 mV s^−1^. The measurements were performed in triplicates and the results are expressed as a mean ± standard deviation.

Cyclic voltammetry in the presence of a redox probe, 2 mg ml^−1^ K_4_[Fe(CN)_6_] in 0.1 M KCl, was used to determine the effective surface area (ESA). CV scans (Fig. S1†) were performed in the potential range from −0.2 V to 0.8 V at a scan rate of 0.1 V s^−1^. ESA was calculated according to the Randles–Sevcik equation:^51,52^

1 *i*_p_ = 2.69 × 10^5^*AD*^1/2^*n*^3/2^*ν*^1/2^*C*

where *i*_p_ is the reduction/oxidation peak current (A), *n* is the number of electrons participating in the redox reaction, *A* is the area of the electrode (cm^2^), *D* is the diffusion coefficient of Fe(CN)_6_^4−^in KCl solution (6.3 × 10^−6^ cm^2^ s^−1^),^53^*C* is the concentration of the Fe(CN)_6_^4−^ in the bulk solution (mol cm^−3^) and *ν* is the scan rate (V s^−1^).

The enlargement factor was calculated basing on the difference in the electroactive surface area (ESA) between CNT film and bare Pt electrode.

Electrochemical impedance characterization of CNT films was performed using PARSTAT 2273 in the three-electrode cell arrangement described above, in 0.1 M KCl solution. The impedance measurements were carried out at a frequency range from 100 mHz to 100 kHz. An AC voltage amplitude of 10 mV was applied during the measurements. Nyquist plots were used to determine the transition point, at which the slope of the curve changes from 45° to 90°, *i.e.* to identify the diffusion-controlled region, and to calculate the diffusion coefficient according to the following formula:^54^

2 *D* = *ωR*^2^

where *D* is the diffusion coefficient (cm^2^ s^−1^), *ω* is the frequency of the transition point (Hz), and *R* is the electrode thickness (cm).

Electrochemical charging/discharging processes were performed by means of potentiostatic and galvanostatic modes. In the potentiostatic (chronoamperometric) experiment, CNT films were subjected to the constant potential of 1.3 V (*vs.* Ag/AgCl) for 30 s (charging) followed by the application of the open circuit potential for 300 s (discharging). The cumulative charge was calculated by integrating current passing through the CNT film by the time of the process. The areal capacitance was calculated by dividing cumulative charge by charging potential and the area of the electrode. Energy density, *E* (W h kg^−1^) and power density, *P* (W kg^−1^) were calculated according to the following formulas:^55,56^3
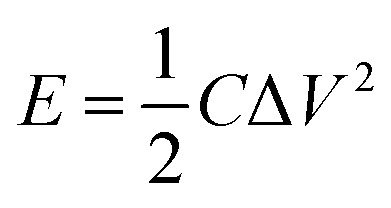
4
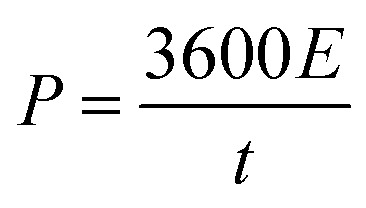
where *C* is the film capacitance (F g^−1^), Δ*V* is the potential difference between charging and discharging processes (V), and *t* is the discharge time (s).

In the galvanostatic (chronopotentiometric) experiment, CNT films were subjected to the current densities of 10 mA cm^−2^. When the potential reached 1.0 V (*vs.* Ag/AgCl), the current was stopped. The process of discharging was monitored until the time when the potential of CNT electrode reached the value of its open circuit potential.

### Reference material

The electrochemical properties of CNT films, such as CSC, impedance and effective surface area, were compared with a reference platinum foil electrode (0.1 mm thickness, 99.9% purity, produced by Mennica-Warsaw, Poland).

## Results and discussion

### Microstructure and composition

**Fig. 1:**
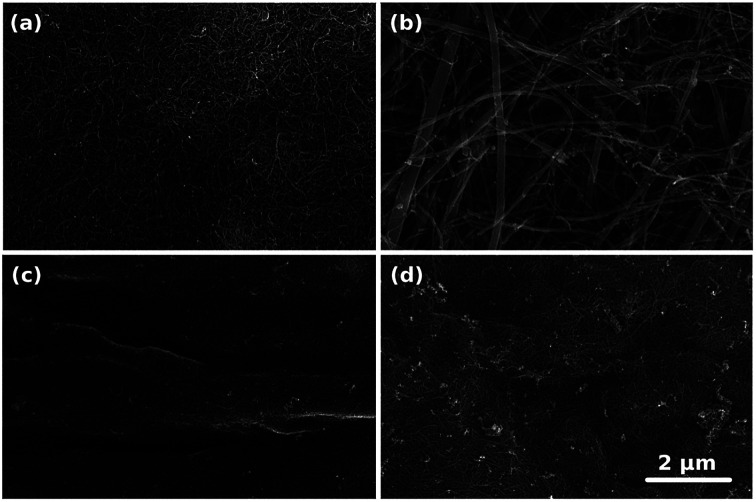
Microstructures of (a) NC7000, (b) carpet, (c) (6,5), (d) (7,6) based CNT films as observed by SEM.

The microstructure of the prepared CNT films was analyzed by SEM (Fig. 1). Macroscopic CNT assemblies made from NC7000 demonstrated a relatively uniform structure (Fig. 1a). SEM micrographs of CNT carpets indicated that the synthesized CNTs possessed considerable polydispersity with larger tube diameters (Fig. 1b).

The microstructure of the prepared CNT films was analyzed by SEM (Fig. 1). Macroscopic CNT assemblies made from NC7000 demonstrated a relatively uniform structure (Fig. 1a). SEM micrographs of CNT carpets indicated that the synthesized CNTs possessed considerable polydispersity with larger tube diameters (Fig. 1b).

**Fig. 2:**
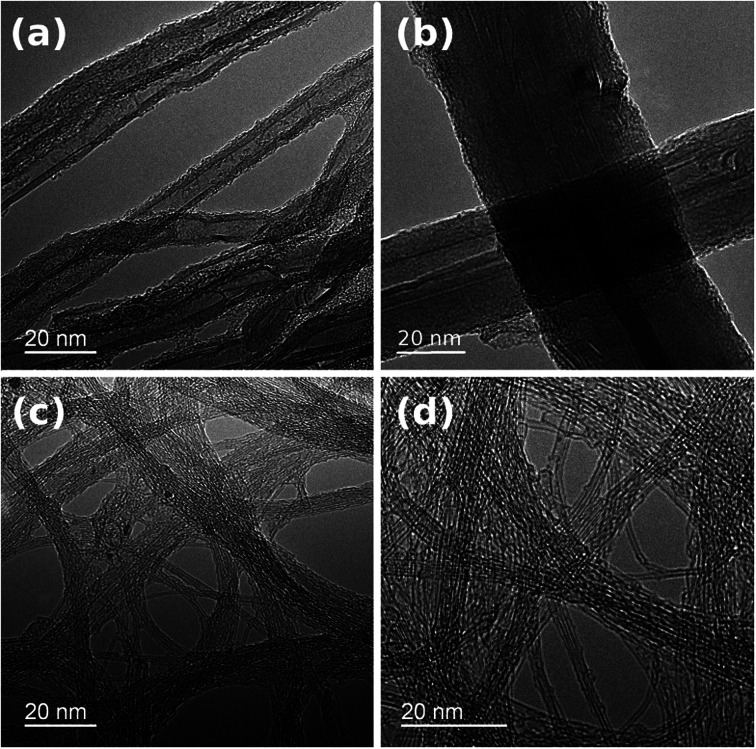
Structure and diameter of individual CNTs in (a) NC7000, (b) carpet, (c) (6,5), (d) (7,6) based CNT films observed by TEM.

The presence of some carbonaceous residue of a non-CNT form was noted, particularly on chirality-sorted samples (Fig. 1c and d). Further investigation by TEM confirmed that NC7000 contained multi-wall CNTs (Fig. 2a). CNT carpets were confirmed again to possess multiple walls and considerable polydispersity in CNT diameter, which was observed to approach 33% (Fig. 2b, standard deviation/average outer diameter). CNT carpet also exhibited reduced graphitization degree relative to other CNT formulations (Fig. 2b). Chirality-sorted (6,5) and (7,6) CNTs were indeed observed to possess single-walls (Fig. 2c and d). The average outer diameter was found to be 10 ± 1 nm, 45 ± 14 nm, 0.80 ± 0.05 nm and 0.85 ± 0.06 nm, respectively.

**Fig. 3:**
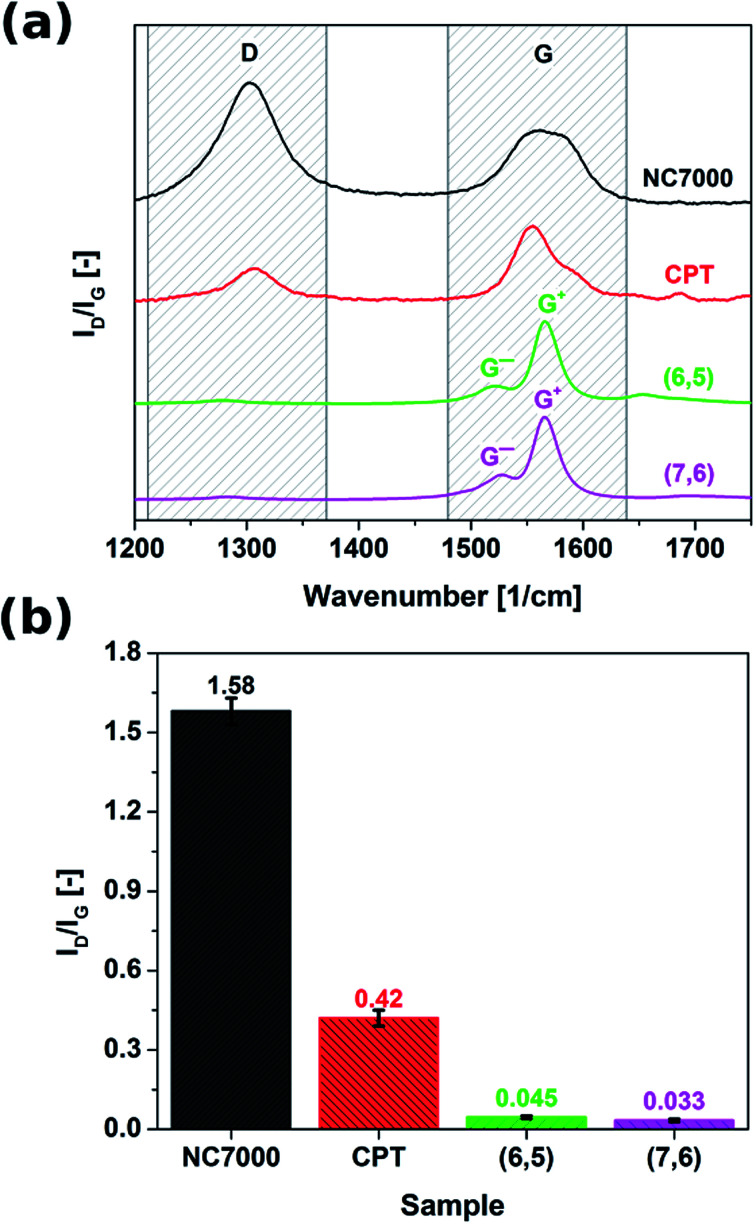
(a) Raman spectra and (b) ratios of intensities of defect-induced band (D) to the band of graphitic lattice (G) of NC7000, carpet, (6,5), (7,6) based CNT films as measured by Raman spectroscopy.

Raman spectroscopy was employed to probe the surface chemistry of all investigated CNT formulations (Fig. 3). Resulting spectra revealed that commercial grade NC7000 CNTs demonstrated significantly elevated sp^3^ contamination relative to other investigated CNT formulations (*I*_D_/*I*_G_ as high as 1.58). The corrugated structure of individual CNTs (Fig. 2a) may be indicative of the abundance of defects and presence of external functional groups. As a consequence of the observed low graphitization levels observed in CNT carpet films, these materials also showed an elevated *I*_D_/*I*_G_ ratio equal to 0.42. Interestingly, chirality-defined (6,5) and (7,6) CNT films were associated with the highest observed purity, with *I*_D_/*I*_G_ ratios of 0.045 and 0.033, respectively. Splitting of G-mode into two components G^−^ and G^+^, out of which the former is of Lorentzian line shape confirms the semiconducting character of the (6,5) and (7,6) CNT films.

More detailed analysis by XPS confirmed the surface composition of the CNT films (Table 1, Fig. S2†). As expected, analysis of the C1s region reveals that the most intense signal comes from C=C component at 284.8 eV. The following components according to available literature and databases can be ascribed to C–C (at 285.5 eV indicative of sp^3^ functionalization), C–O (at 286.7 eV, also standing for C–OH component), C=O (287.8 eV) and broad COOH component (288.9 eV). The last one (291 eV) shall be identified as π–π* shake-up feature. Chirality enriched films mostly composed of (6,5) and (7,6) CNTs had much higher sp^2^/sp^3^ ratio, what is in accordance with high degree of structural perfection confirmed by Raman spectroscopy.

**Table 1:** XPS analysis results

Component	Concentration of species in a sample [%]			
	NC7000	CPT	(6,5)	(7,6)
C=C	63.86	63.56	66.92	66.36
C–C	17.36	18.98	11.49	11.32
C–O	7.42	7.25	8.27	9.22
C=O	3.41	2.3	3.62	3.53
O–C=O	3.42	3.15	4.26	4.52
π–π	4.53	4.75	5.44	5.04
sp^2^/sp^3^	3.68	3.35	5.82	5.86

**Fig. 4:**
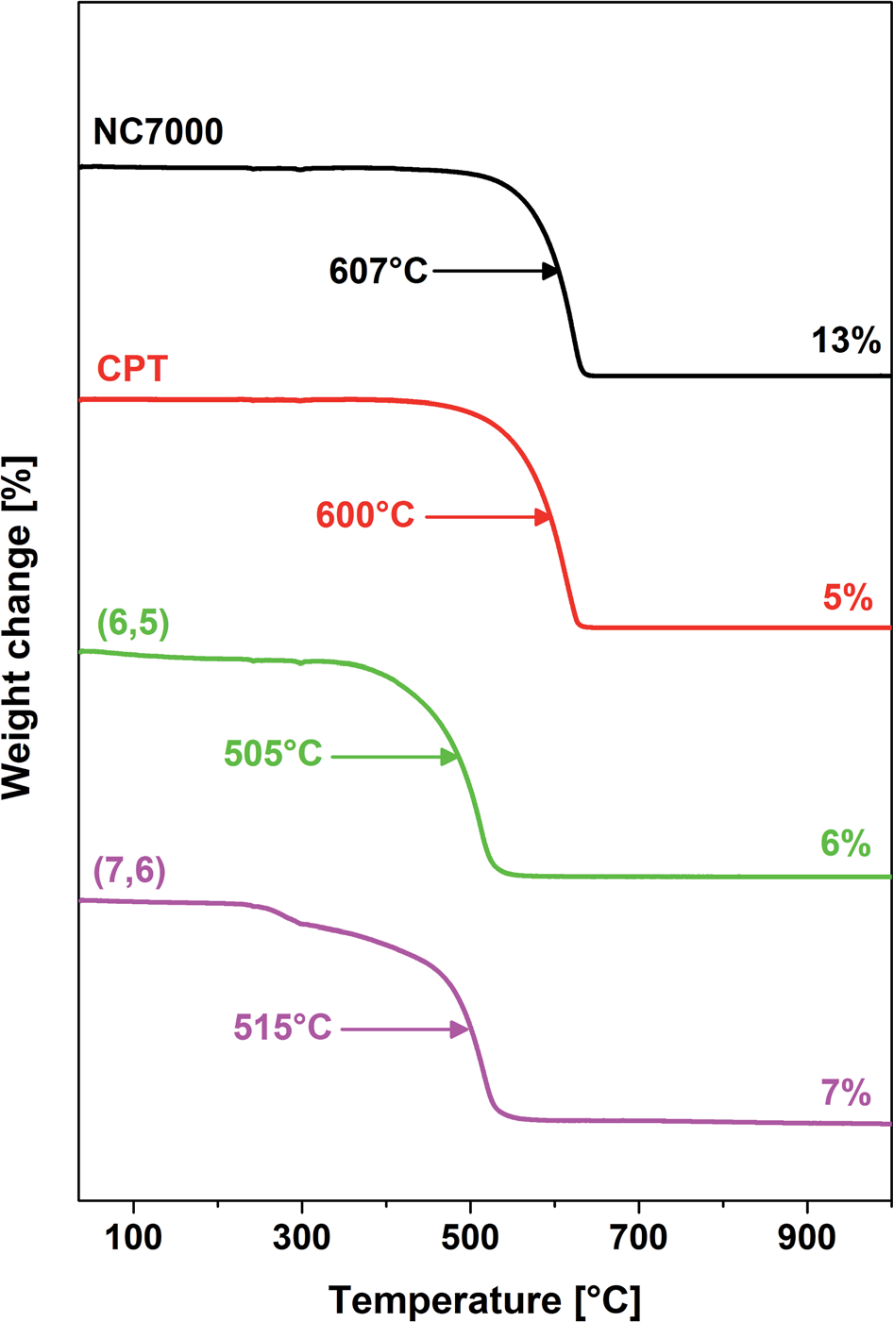
Thermograms of NC7000-, carpet-, (6,5)-, (7,6)-based CNT films as measured by TGA. The arrows show the temperature of maximum rate of decomposition, whereas the values on the right give the amount of residue left after combustion of the samples.

Thermogravimetric analysis (Fig. 4) revealed that films formed from single-wall CNTs as expected were associated with a reduced thermal stability relative to multi-wall CNT films.^57^ Furthermore, NC7000- and carpet-based multi-wall CNT films decomposed at temperatures of ∼600 °C whereas chirality-predominant (6,5)- and (7,6)-based single-wall CNT films decomposed at reduced temperatures, ∼500 °C. Furthermore, NC7000 CNTs had the highest content of residual catalyst (13%) relative to other CNT film formulations (5–7%) (Fig. 4).

**Fig. 5:**
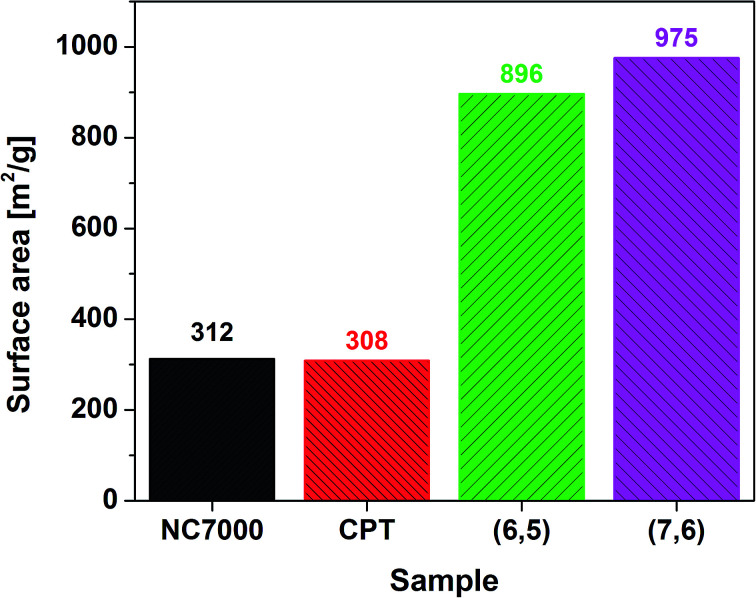
BET surface area calculated by means of nitrogen adsorption.

Nitrogen adsorption experiments showed that the CNT films have a well developed surface area (Fig. 5). Multi-wall based NC7000 and CPT CNT films had on average three times lower surface area than (6,5) and (7,6) CNT films built from small diameter single-wall CNTs. The measured values are within the theoretical surface area window calculated for CNTs of various number of walls and diameter.^58^ Because of the fact that the surface is much more developed for (6,5) and (7,6) enriched CNT films more adventitious contaminants can adsorb on the surface. As a consequence, the content of adulterants for these samples as estimated by XPS is probably overestimated.

### Electrochemical properties

**Fig. 6:**
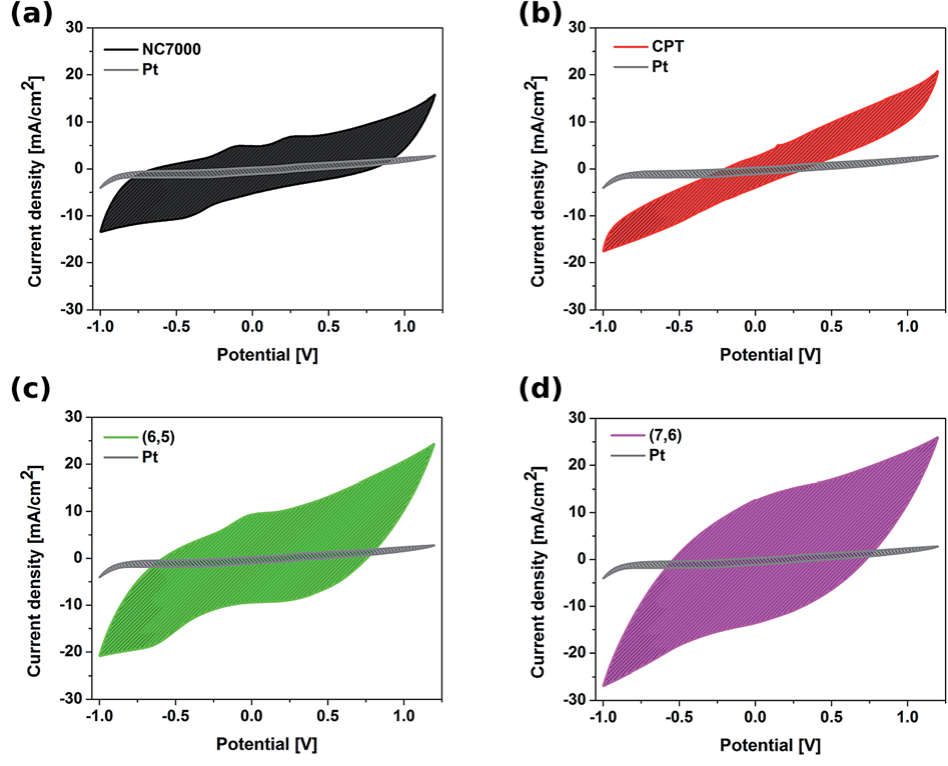
Cyclic voltammograms collected in 0.1 M KCl at a scan rate of 0.1 V s^−1^ of all experimental CNT film formulations (a) NC7000, (b) carpet, (c) (6,5) CNTs, (d) (7,6) CNTs overlaid onto the Pt foil reference. Shading represents the area that was used to calculate charge storage capacities (CSC).

The electrochemical behavior of CNT films was studied by cyclic voltammetry (CV) as well as electrochemical impedance spectroscopy (EIS), and compared with the electrochemical behavior of a platinum electrode. CVs recorded for different types of CNT working electrodes (Fig. 6) show the discrepancies in electrochemical behavior of CNT films composed of NC7000, carpets and chirality-sorted CNTs.

The featureless CV recorded for unsorted CNT carpet films was as a result of the wide distribution of the structure of CNTs, which is in accordance with electron microscopy studies. Because of the fact that this film is composed of CNTs having structural variations, such as length, diameter and chirality, the CV shows the average of many closely spaced peaks representing electron transfer into each CNT.^59^ Resulting CVs of (7,6)-based CNT film also did not reveal evident reversible redox processes occurring on the electrode surface. This was however, not observed in the CVs of sorted CNT (6,5) film electrodes, for which the anodic (0 V) and cathodic (−0.6 V) peaks, characteristic for this type of structure, were observed. The presence of two weak oxidation peaks (−0.10 V and 0.25 V) and one reduction peak (−0.40 V) in the CV of NC7000-based CNT film confirmed the abundance of functional groups on the surface, what is in accordance with results from Raman spectroscopy.

Such features are also evident but to a lesser extent in the case of CPT and (6,5) CNTs, due to their more crystalline structure than NC7000. The results are in agreement with the previous literature reports showing the strong dependency of the density of states on CNT diameter, length, chirality and type,^60^ leading to the significant effect of geometrical properties of CNT on their electronic properties.^61^

**Fig. 7:**
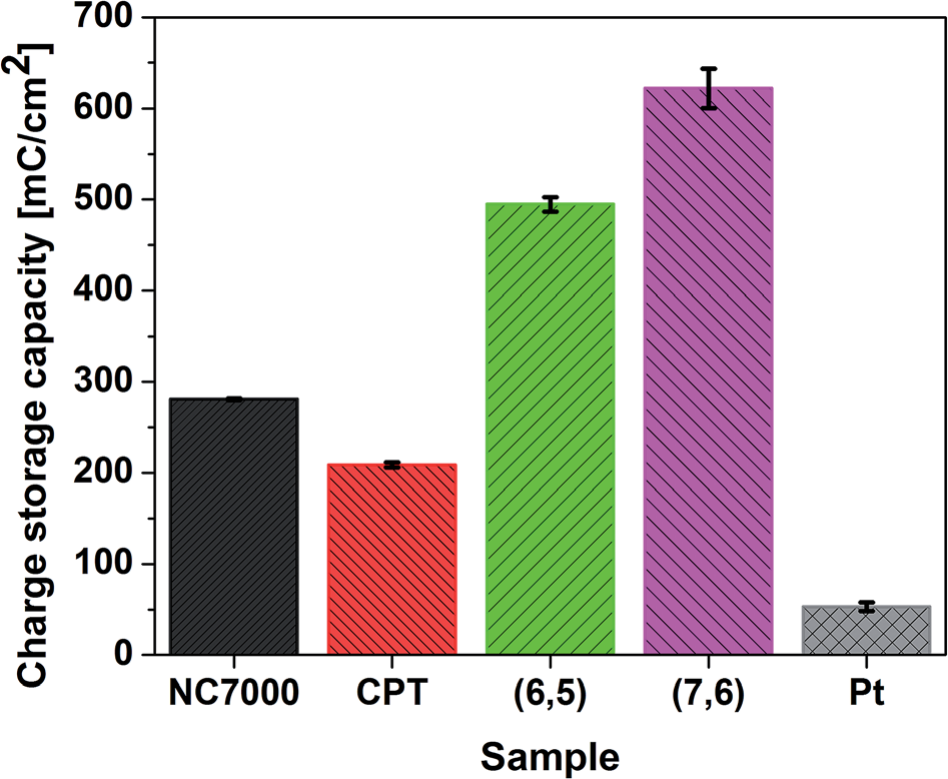
Charge storage capacities of all experimental CNT film formulations and Pt calculated by the integration of corresponding CV curves recorded in 0.1 M KCl in the potential range from −1.0 V to 1.2 V (*vs.* Ag/AgCl) at a scan rate of 0.1 V s^−1^.

CVs recorded for all CNT films exhibit double layer capacitance behavior, which is represented by large areas under corresponding CV curves.^59,62,63^ Consequently, these CNT films were associated with significantly high values of CSC (Fig. 7), defined as the charge passing through the electrode and calculated as the time integral of the corresponding CV curve. The highest CSC (621.91 ± 21.61 mC cm^−2^) was observed with (7,6) CNT film formulations, which were composed of high quality and low diameter CNTs; this measured charge storage capacity was 25% higher than for (6,5) CNTs and 12 times higher than for pristine Pt.

This is an exceptionally high CSC, significantly higher than for many other CNT-based high charge storage capacity materials reported in the literature, including iridium oxide/CNT (101.2 mC cm^−2^),^64^ CNT yarns (98.6 mC cm^−2^)^65^ and PEDOT/MWCNT (202.9 mC cm^−2^).^66^ The high value of CSC is caused by the regular structure and low defect number of employed single-wall CNTs, which indicated the improved electrochemical and electroanalytical parameters relative to multi-wall CNTs.^67^ The enhanced double layer capacitance behavior was additionally confirmed by collecting CV curves at different scan rates (Fig. S3a–d†) and plotting the current *vs.* square root of scan rate plot (Fig. S4†). The linear character of this dependency in case of CPT (*R*^2^ > 0.99) and NC7000 (*R*^2^ > 0.98) confirm that electrochemical processes for these materials are controlled by the diffusion. The decreased value of *R*^2^ for chirality-sorted (6,5) CNT (*R*^2^ > 0.97) and especially for (7,6) CNT (*R*^2^ > 0.91) show that the process of charge transfer is not fully diffusion-controlled, but is most probably accompanied by faradaic reaction.

Moreover, as the result of the process of sorting and the monodispersity of films formulated with both (6,5) and (7,6) CNTs, these materials demonstrated enhanced electrochemical performance as well as significantly increased effective surface area (ESA). The effective surface area was determined using a redox probe (2.5 mol dm^−3^ K_4_[Fe(CN)_6_] in 0.1 M KCl) solution and estimated according to the Randles–Sevcik equation. Table 2 shows that all experimental CNT film formulations possessed an increased ESA when compared to a bare Pt electrode. The enlargement factor was determined as 4.7 for NC7000. 6.8 for CPT, and more than 8 and 10 for chiral (6,5) and (7,6) CNT films, respectively. These results confirm that the development of the physical surface area determined by nitrogen adsorption experiments is accompanied with the development in electrochemically effective surface, and indicate chiral CNT films to outperform both unsorted NC7000 and CPT films. The evolution of effective surface area can be considered as particularly advantageous in the design of energy storage devices, especially electric double layer capacitors, where both high CSC and large ESA are desired^68,69^ and necessary for design of ultrathin film electrodes.^46^

**Table 2:** Effective surface area and enlargement factors (determined as the increase in electroactive surface area with respect to bare Pt microelectrode), calculated basing on the characteristic redox peaks of K_4_[Fe(CN)_6_] collected on CNT films during cyclic voltammetry in 0.1 M KCl in the potential range from −0.2 V to 0.8 V (*vs.* Ag/AgCl) at 0.1 V s^−1^ scan rate

	Effective surface area (cm^2^)	Enlargement factor
Pt	0.628	1
NC7000	2.945	4.7
CPT	4.250	6.8
(6,5)	5.178	8.2
(7,6)	6.697	10.7

**Fig. 8:**
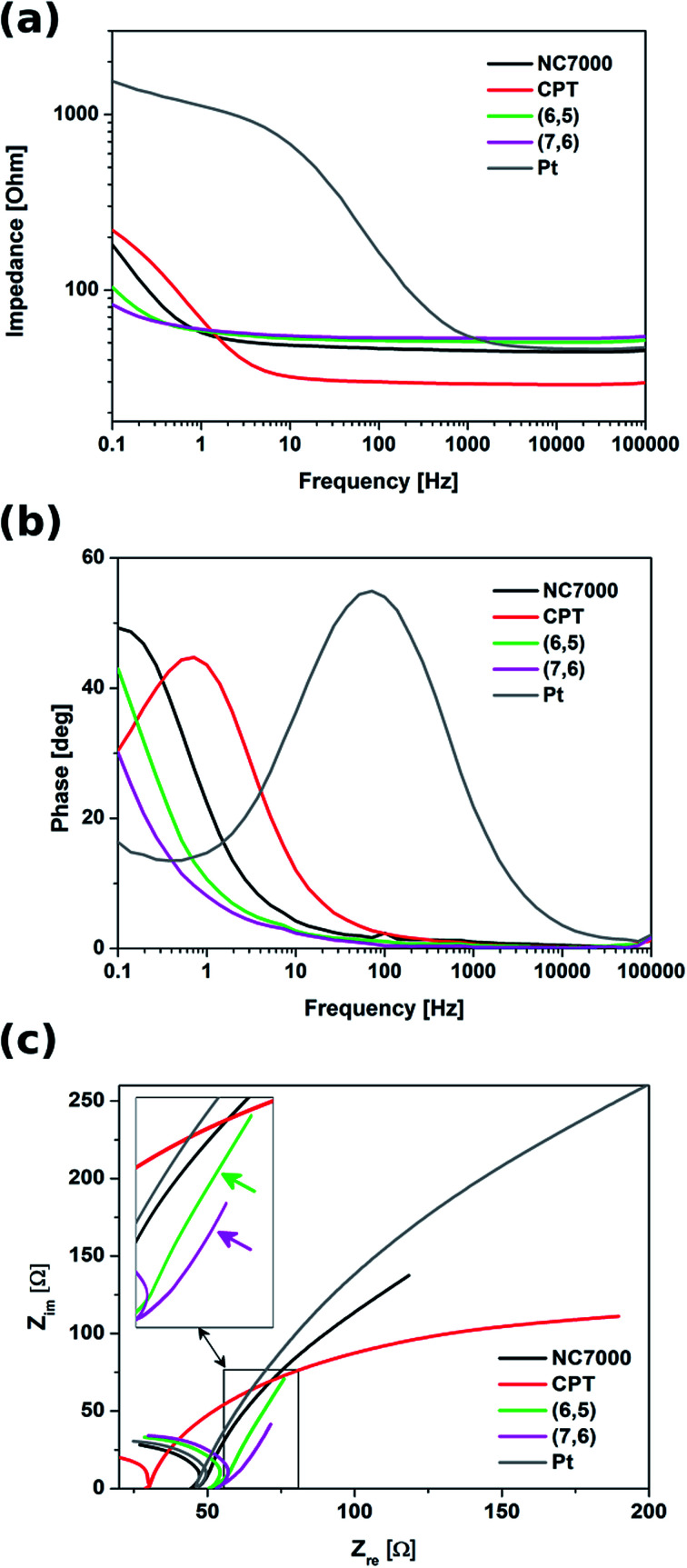
Bode plots showing (a) impedance modules and (b) phase profiles as a function of frequency, as well as (c) Nyquist plots for different types of CNT films (NC7000, carpet, (6,5) and (7,6) CNTs) and Pt. The inset indicates the linear part in the low frequency region characteristic for the ideal capacitor.

Electrochemical impedance spectroscopy indicated that all experimental CNT film formulations exhibited significantly lower impedance relative to control Pt electrode, especially in the low frequency region (below 1 kHz) (Fig. 8). In this region these were chirality-sorted CNTs that outperformed both unsorted CNTs and bare Pt with respect to low impedance. With frequencies higher than 1 kHz, the impedance spectra of Pt were observed to decrease to a value comparable with that of investigated CNT film formulations, as well as the impedance of NC7000 and CPT decreased to the values lower than for (6,5) and (7,6) CNTs. This discrepancy can be explained by the fact that the unsorted CNTs exhibit strong diffusion-controlled capacitive behavior, just as it was shown by the analysis of CVs collected at different scan rates. Due to their mixed electron transport mechanism, the impedance profile of chirality-sorted CNTs must differ from the behavior of ideal capacitor,^70^ manifested by almost flat line in the whole frequency range.

Although CNT carpet films demonstrated an impedance trend similar to that of Pt, this film formulation was observed to possess the lowest impedance profile in the high frequency range down to 1 Hz. The plots of phase *vs.* frequency (Fig. 8b) indicated that from the point of view of the mechanism of charge transfer, CNT film formulated from CNT carpets most resembled Pt relative to other film formulation investigated in this study, and hence did not exhibit enhanced capacitive behavior. This type of behavior, however, was observable in the Nyquist plots (Fig. 8c) of both sorted CNT films employed in this study, which possessed vertical line forms in the low frequency region, characteristic for ideal capacitors.^71,72^ The capacitive behavior was particularly clear for (7,6) CNT films, where the system transition to a more capacitive behavior was observed at the frequency of 0.5 Hz. According to,^54^ the frequency at the transition point at which the slope of the curve changes from 45° to 90°, together with film thickness, can be used to determine the diffusion coefficient of electrolyte ions. In case of (7,6) CNT films, the diffusion coefficient of electrolyte ions was calculated to be equal to 1.57 × 10^−5^ cm^2^ s^−1^. This value is significant, especially when compared with the diffusion coefficients determined for other highly capacitive materials, *e.g.* activated carbon (22.0 × 10^−9^ cm^2^ s^−1^)^73^ and polymeric nickel complexes (4.3 × 10^−7^ cm^2^ s^−1^).^74^ The enhanced diffusion of ions within the material is supposed to decrease the Warburg impedance,^75^ which has the positive impact on the power density of the capacitor.^74^

**Fig. 9:**
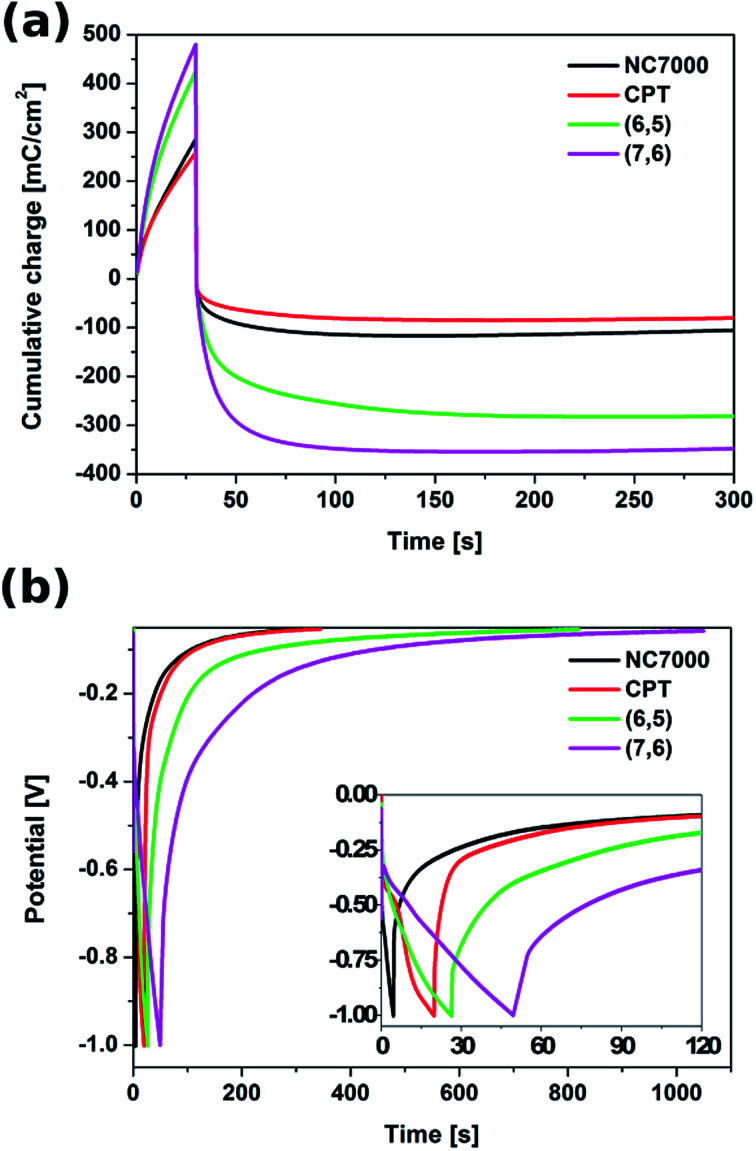
Cumulative charge accumulated and released during (a) chronoamperometric charging/discharging process and (b) chronopotentiometric curve of charging/discharging process of all experimental CNT film formulations.

The energy storage performance of the all experimental CNT film formulations was studied in both potentiostatic and galvanostatic modes. In the potentiostatic (chronoamperometric) mode (Fig. S5†), CNT films were subjected to a constant potential of 1.3 V (*vs.* Ag/AgCl) for 30 s (charging) and to an open circuit potential for the time needed to reach a fully discharged state (300 s). The cumulative charge passing through the CNT films during this charging/discharging process, presented in Fig. 9a, showed that the most effective capacitance behavior was exhibited by the chiral (7,6) CNT film. In only 30 s of potential application, the cumulative charge reached 495 mC cm^−2^.

During 30 s of the discharging process, (7,6) CNT film was able to release the charge of 309 mC cm^−2^ and the full discharge was observed after 300 s, when the charge of 341 mC cm^−2^ was released, giving the total areal capacitance of 262 mF cm^−2^ (Table 3). This film exhibited also superior behavior in terms of mass capacitance (80.6 F g^−1^), energy density (16.5 W h kg^−1^) as well as power density (220.3 W kg^−1^). The capacitive properties of (7,6) CNT film, especially its areal capacitance, are remarkable when compared with other materials described as effective (super)capacitors, *e.g.* C-doped TiO_2_ nanotube arrays (12 mF cm^−2^),^76^ polypyrrole/Au scaffolds (37 mF cm^−2^),^77^ CNT/MnO_2_ composites (52 mF cm^−2^),^78^ activated carbon/PVDF/graphite/MWCNT composites (90.7 mF cm^−2^)^45^ and polyaniline/polyoxometalate hybrid nanocomposites (195 mF cm^−2^).^79^

**Table 3:** Accumulated charge, areal capacitance, mass capacitance, energy and power densities calculated basing on the corresponding chronoamperometric discharging curves

Parameter	Sample			
	NC7000	CPT	(6,5)	(7,6)
Accumulated charge (mC cm^−2^)	101	74	275	341
Areal capacitance (mF cm^−2^)	78	57	212	262
Mass capacitance (F g^−1^)	17.5	11.4	73.6	80.6
Energy density (W h kg^−1^)	3.0	2.2	13.6	16.5
Power density (W kg^−1^)	40.0	28.7	181.5	220.3

### Charging/discharging characteristics

With the accumulated charge of 275 mC cm^−2^ (212 mF cm^−2^), also chiral (6,5) CNT film could be treated as a high capacity material. The unsorted NC7000 and CPT films are characterized with the charge capacities of 101 mC cm^−2^ (78 mF cm^−2^) and 74 mC cm^−2^ (57 mF cm^−2^), respectively, still remarkable when compared with literature.

The galvanostatic test was conducted with the application of a charging current of 10 mA cm^−2^ and a zero discharging current. The corresponding chronopotentiometric curves, shown in Fig. 9b, confirm that chiral CNT films are able to accumulate charge and release it in a prolonged time. Also in this test chiral CNTs proved to resemble the behavior of ideal capacitors relative to unsorted CNT film formulations.^45,80^

## Conclusions

In summary, CNT films of various number of walls, diameter, chirality and surface chemistry were formulated and evaluated with respect to their electrochemical performance. Comparison with other electrode materials reported in the literature indicated that chirality-defined pristine single-wall CNT films (7,6 in particular) had excellent charge storage capacity (up to 621.91 mC cm^−2^), areal capacitance (262 mF cm^−2^), very high effective surface area and preferential charge/discharge characteristics. High degree of crystallinity of both (6,5) and (7,6) enriched s-SWCNT films and significantly reduced presence of extraneous impurities makes such ensembles (as compared with polydisperse mixtures of MWCNTs of moderate quality and significant content of metallic CNTs) very attractive for charge storage applications. They could already offer competitive advantage over traditionally employed materials, especially the materials applied as supercapacitors. We believe that with further progress in the sorting of CNTs (and manufacture of macroscopic ensembles from such materials), we will not only be able to better understand the nature of carbon nanomaterials, but it will enable the design of CNT devices with properties tailored for a specific application. The results of this study demonstrate that sorted CNT macroassemblies show great potential for energy storage technologies to be discharged from R&D laboratories to the real life.

## Conflicts of interest

The authors declare no conflicts of interest.

## Supplementary Material

RA-008-C8RA03963A-s001

## References

[cit1] Hong S., Myung S. (2007). Nat. Nanotechnol..

[cit2] Brady G. J., Way A. J., Safron N. S., Evensen H. T., Gopalan P., Arnold M. S. (2016). Sci. Adv..

[cit3] Geim A. K., Novoselov K. S. (2007). Nat. Mater..

[cit4] Yu M.-F., Lourie O., Dyer M. J., Moloni K., Kelly T. F., Ruoff R. S. (2000). Science.

[cit5] Sinnott S. B., Andrews R. (2001). Crit. Rev. Solid State Mater. Sci..

[cit6] Lee C., Wei X., Kysar J. W., Hone J. (2008). Science.

[cit7] Pop E., Mann D., Wang Q., Goodson K., Dai H. (2006). Nano Lett..

[cit8] Koziol K. K., Janas D., Brown E., Hao L. (2017). Phys. E.

[cit9] Balandin A. A., Ghosh S., Bao W., Calizo I., Teweldebrhan D., Miao F., Lau C. N. (2008). Nano Lett..

[cit10] Janas D., Czechowski N., Krajnik B., Mackowski S., Koziol K. K. (2013). Appl. Phys. Lett..

[cit11] Tang Z. K., Zhang L., Wang N., Zhang X. X., Wen G. H., Li G. D., Wang J. N., Chan C. T., Sheng P. (2001). Science.

[cit12] Legoas S. B., Coluci V. R., Braga S. F., Coura P. Z., Dantas S. O., Galvão D. S. (2004). Nanotechnology.

[cit13] Tombros N., Jozsa C., Popinciuc M., Jonkman H. T., van Wees B. J. (2007). Nature.

[cit14] Iijima S. (1991). Nature.

[cit15] Novoselov K. S., Geim A. K., Morozov S. V., Jiang D., Zhang Y., Dubonos S. V., Grigorieva I. V., Firsov A. A. (2004). Science.

[cit16] Janas D., Koziol K. K. (2013). Carbon.

[cit17] Sumita S., Syed Z. A., Prasanta K. G., Guofang Z., John R., James A. C., William I. M., Julian W. G., Florin U. (2010). Nanotechnology.

[cit18] Fang Y., Hester J. G. D., Su W., Chow J. H., Sitaraman S. K., Tentzeris M. M. (2016). Sci. Rep..

[cit19] Dejan T., Aleksandar M., Marijana M., Djordje J., Radoš G., Iva S., Marko S. (2015). 2D Materials.

[cit20] Stevens R. M. (2009). Mater. Today.

[cit21] https://www.zyvextech.com/

[cit22] Anguita J. V., Ahmad M., Haq S., Allam J., Silva S. R. P. (2016). Sci. Adv..

[cit23] Mathur R. B. , Singh B. P. and Pande S., Carbon Nanomaterials: Synthesis, Structure, Properties and Applications, CRC Press Taylor & Francis Group, 2017

[cit24] Avery A. D., Zhou B. H., Lee J., Lee E.-S., Miller E. M., Ihly R., Wesenberg D., Mistry K. S., Guillot S. L., Zink B. L., Kim Y.-H., Blackburn J. L., Ferguson A. J. (2016). Nat. Energy.

[cit25] Charnvanichborikarn S., Shin S. J., Worsley M. A., Kucheyev S. O. (2012). Appl. Phys. Lett..

[cit26] Janas D., Boncel S., Koziol K. K. K. (2014). Carbon.

[cit27] Lekawa-Raus A., Patmore J., Kurzepa L., Bulmer J., Koziol K. (2014). Adv. Funct. Mater..

[cit28] Lekawa-Raus A., Gizewski T., Patmore J., Kurzepa L., Koziol K. K. (2017). Scr. Mater..

[cit29] Xiong Z., Yun S. Y., Jin H.-J. (2013). Materials.

[cit30] Landi B. J., Ganter M. J., Cress C. D., DiLeo R. A., Raffaelle R. P. (2009). Energy Environ. Sci..

[cit31] Kucinskis G., Bajars G., Kleperis J. (2013). J. Power Sources.

[cit32] Wang H., Cui L.-F., Yang Y., Sanchez Casalongue H., Robinson J. T., Liang Y., Cui Y., Dai H. (2010). J. Am. Chem. Soc..

[cit33] Pan H., Li J., Feng Y. (2010). Nanoscale Res. Lett..

[cit34] Peng C., Zhang S., Jewell D., Chen G. Z. (2008). Prog. Nat. Sci..

[cit35] Ke Q., Wang J. (2016). J. Materiomics.

[cit36] Liu C., Fan Y. Y., Liu M., Cong H. T., Cheng H. M., Dresselhaus M. S. (1999). Science.

[cit37] Dillon A. C., Jones K. M., Bekkedahl T. A., Kiang C. H., Bethune D. S., Heben M. J. (1997). Nature.

[cit38] Dimitrakakis G. K., Tylianakis E., Froudakis G. E. (2008). Nano Lett..

[cit39] Dai L., Chang D. W., Baek J.-B., Lu W. (2012). Small.

[cit40] Wang Y., Tang S., Vongehr S., Ali Syed J., Wang X., Meng X. (2016). Sci. Rep..

[cit41] Chen Y., Du L., Yang P., Sun P., Yu X., Mai W. (2015). J. Power Sources.

[cit42] Ferrero G. A., Sevilla M., Fuertes A. B. (2017). Sustainable Energy Fuels.

[cit43] Liao C.-Y., Kuok F.-H., Chen C.-W., Hsu C.-C., Chen J.-Z. (2017). J. Energy Storage.

[cit44] Li X., Zhao T., Wang K., Yang Y., Wei J., Kang F., Wu D., Zhu H. (2011). Langmuir.

[cit45] Shen C., Wang X., Zhang W., Kang F. (2011). J. Power Sources.

[cit46] Xu Y., Lin Z., Huang X., Liu Y., Huang Y., Duan X. (2013). ACS Nano.

[cit47] Hughes M., Chen G. Z., Shaffer M. S. P., Fray D. J., Windle A. H. (2002). Chem. Mater..

[cit48] Singh C., Shaffer M. S. P., Windle A. H. (2003). Carbon.

[cit49] Janas D., Rdest M., Koziol K. (2017). Mater. Des..

[cit50] Krukiewicz K., Zak J. K. (2014). J. Mater. Sci..

[cit51] Wen M., Liu H., Zhang F., Zhu Y., Liu D., Tian Y., Wu Q. (2009). Chem. Commun..

[cit52] Bard A. J. and Faulkner L. R., Electrochemical Methods: Fundamentals and Applications, John Wiley and Sons, New York, 2nd edn, 2001

[cit53] Stevens N. P. C., Rooney M. B., Bond A. M., Feldberg S. W. (2001). J. Phys. Chem. A.

[cit54] Katherine L. V. A., John K. M., Song L., Guang F., Suresh M. C., Eugene M., Pasquale F. F., Peter T. C., Sheng D., Yury G. (2014). J. Phys.: Condens. Matter.

[cit55] Tomko T., Rajagopalan R., Lanagan M., Foley H. C. (2011). J. Power Sources.

[cit56] He D., Niu J., Dou M., Ji J., Huang Y., Wang F. (2017). Electrochim. Acta.

[cit57] Kim Y. A., Muramatsu H., Hayashi T., Endo M., Terrones M., Dresselhaus M. S. (2004). Chem. Phys. Lett..

[cit58] Peigney A., Laurent C., Flahaut E., Bacsa R. R., Rousset A. (2001). Carbon.

[cit59] Liu C. y., Bard A. J., Wudl F., Weitz I., Heath J. R. (1999). Electrochem. Solid-State Lett..

[cit60] Kim S. N., Rusling J. F., Papadimitrakopoulos F. (2007). Adv. Mater..

[cit61] Fagan-Murphy A., Kataria S., Patel B. A. (2016). J. Solid State Electrochem..

[cit62] Chen J. H., Li W. Z., Wang D. Z., Yang S. X., Wen J. G., Ren Z. F. (2002). Carbon.

[cit63] Chung S., Kang H., Ocon J. D., Lee J. K., Lee J. (2015). Curr. Appl. Phys..

[cit64] Carretero N. M., Lichtenstein M. P., Pérez E., Cabana L., Suñol C., Casañ-Pastor N. (2014). Acta Biomater..

[cit65] Jiang C., Li L., Hao H. (2011). IEEE Trans. Neural Syst. Rehabil. Eng..

[cit66] Zhou H., Cheng X., Rao L., Li T., Duan Y. Y. (2013). Acta Biomater..

[cit67] Jiang H., Du C., Zou Z., Li X., Akins D. L., Yang H. (2009). J. Solid State Electrochem..

[cit68] Afzal A., Abuilaiwi F. A., Habib A., Awais M., Waje S. B., Atieh M. A. (2017). J. Power Sources.

[cit69] Frackowiak E., Béguin F. (2001). Carbon.

[cit70] Cesiulis H. , Tsyntsaru N., Ramanavicius A. and Ragoisha G., in Nanostructures and Thin Films for Multifunctional Applications: Technology, Properties and Devices, ed. I. Tiginyanu, P. Topala and V. Ursaki, Springer International Publishing, Cham, 2016, pp. 3–42, 10.1007/978-3-319-30198-3_1

[cit71] Zhang Z., Xiao F., Qian L., Xiao J., Wang S., Liu Y. (2014). Adv. Energy Mater..

[cit72] Selvakumar M., Krishna Bhat D., Manish Aggarwal A., Prahladh Iyer S., Sravani G. (2010). Phys. B.

[cit73] Liu X., Wang Y., Zhan L., Qiao W., Liang X., Ling L. (2011). J. Solid State Electrochem..

[cit74] Alekseeva E. V., Chepurnaya I. A., Malev V. V., Timonov A. M., Levin O. V. (2017). Electrochim. Acta.

[cit75] Liu X.-m., Zhang R., Zhan L., Long D.-h., Qiao W.-m., Yang J.-h., Ling L.-c. (2007). New Carbon Mater..

[cit76] Zhang J., Gao B., Gan Q., Xia J., Cao Y., Ma Y., Wang J., Huo K. (2014). Chem. Rapid Commun..

[cit77] Lu P., Ohlckers P., Chen X. Y. (2016). J. Phys.: Conf. Ser..

[cit78] Higgins T. M., McAteer D., Coelho J. C. M., Sanchez B. M., Gholamvand Z., Moriarty G., McEvoy N., Berner N. C., Duesberg G. S., Nicolosi V., Coleman J. N. (2014). ACS Nano.

[cit79] Gómez-Romero P., Chojak M., Cuentas-Gallegos K., Asensio J. A., Kulesza P. J., Casañ-Pastor N., Lira-Cantú M. (2003). Electrochem. Commun..

[cit80] Shen J., Liu A., Tu Y., Foo G., Yeo C., Chan-Park M. B., Jiang R., Chen Y. (2011). Energy Environ. Sci..

