# Supplementary material for: Chirality-sorted carbon nanotube films as high capacity electrode materials
Source: RSC Adv. 2018 Aug 30;8(53):30600–9. doi: 10.1039/c8ra03963a (PMC9085468; doi:10.1039/c8ra03963a)
Supplement: RA-008-C8RA03963A-s001 [file RA-008-C8RA03963A-s001.pdf]

Electronic Supplementary Material (ESI) for RSC Advances.
This journal is © The Royal Society of Chemistry 2018

Electronic supplementary information

Chirality-sorted carbon nanotube films as high capacity electrode materials

Katarzyna Krukiewicz[b,c], Maciej Krzywiecki[d], Manus J.P. Biggs[b], Dawid Janas[a,*]

[a] *Department of Organic Chemistry, Bioorganic Chemistry and Biotechnology, Silesian University of Technology, B. Krzywoustego 4, 44-100 Gliwice, Poland*

[b] *CÚRAM - Centre for Research in Medical Devices, National University of Ireland, Galway, 118 Corrib Village, Galway, Ireland*

[c] *Department of Physical Chemistry and Technology of Polymers, Silesian University of Technology, M. Strzody 9, 44-100 Gliwice, Poland*

[d] *Institute of Physics – CSE, Silesian University of Technology, Konarskiego 22B, 44-100 Gliwice, Poland*

[*]Corresponding author. Tel: +48 32 2371082. E-mail address: dawid.janas@polsl.pl (D. Janas)

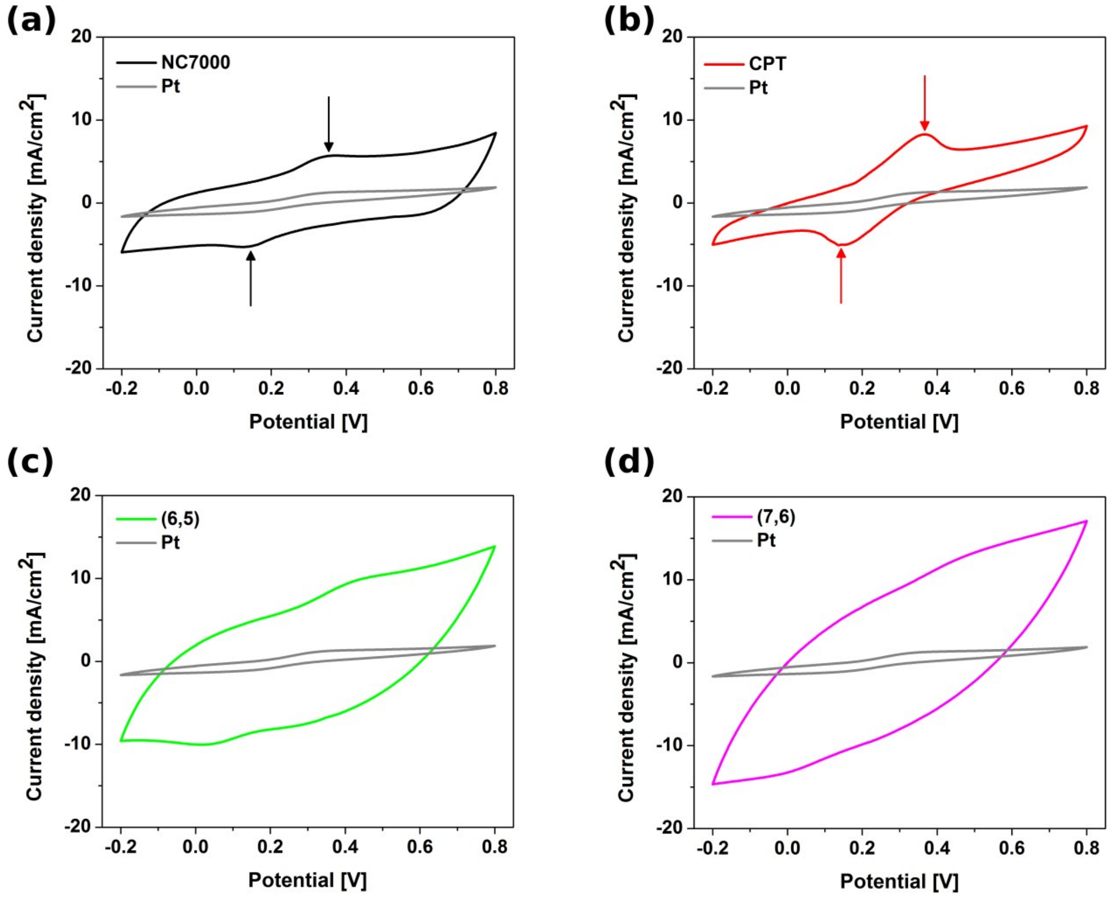

Figure S1 Cyclic voltammograms recorded in the presence of $K_4[Fe(CN)_6]$ on different types of CNT films (a) NC7000, (b) carpet, (c) (6,5) CNTs, (d) (7,6) CNTs overlaid onto the Pt foil reference.

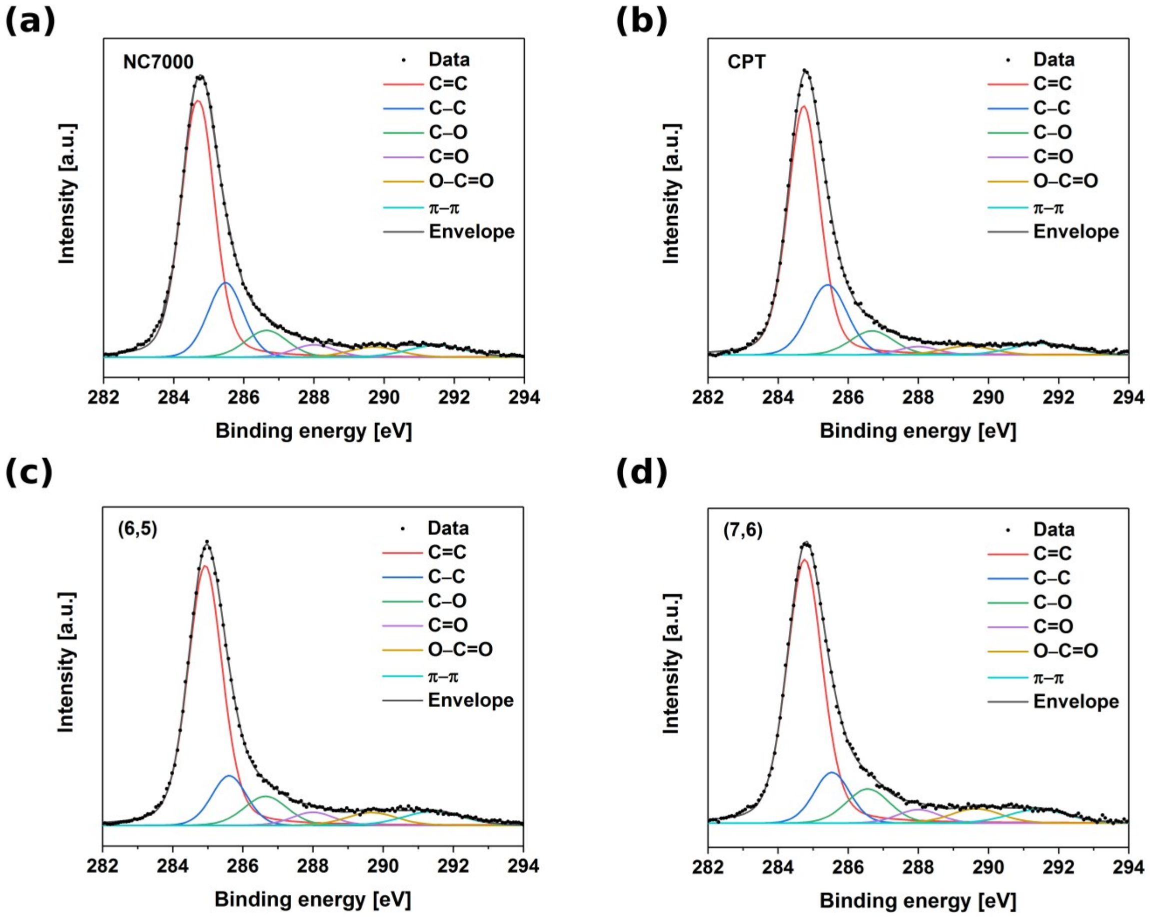

Figure S2 Surface analysis by XPS of all experimental CNT film formulations (a) NC7000, (b) carpet, (c) (6,5) CNTs, (d) (7,6) CNTs.

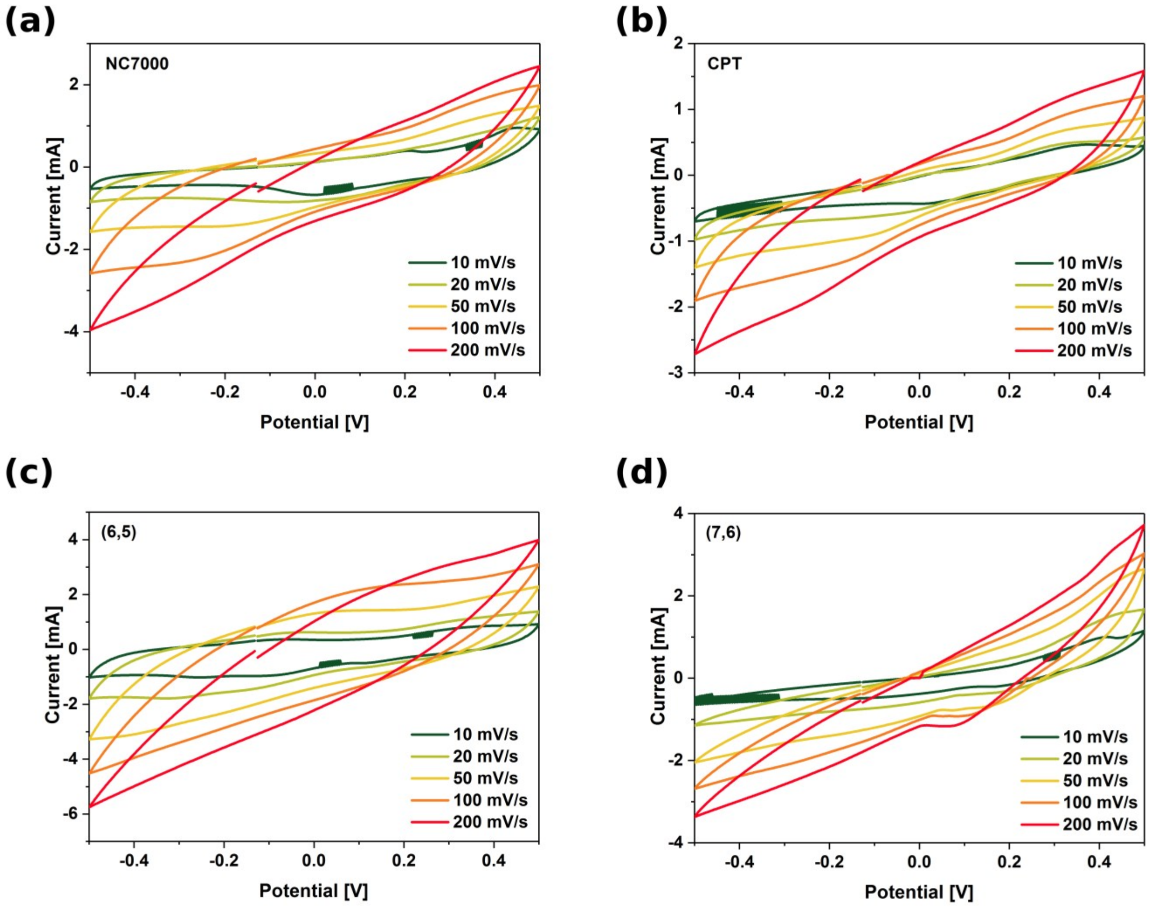

Figure S3 Cyclic voltammograms collected in 0.1 M KCl at a scan rate of 10 mV/s, 20 mV/s, 50 mV/s, 100 mV/s and 200 mV/s of all experimental CNT film formulations (a) NC7000, (b) carpet, (c) (6,5) CNTs, (d) (7,6) CNTs.

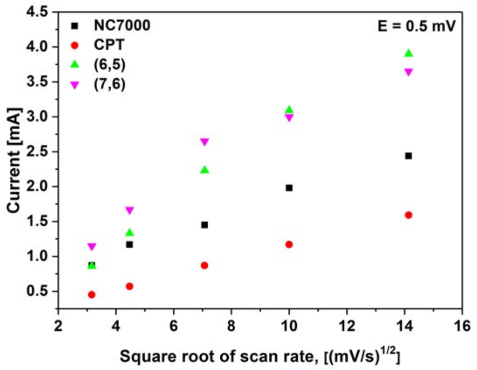

Figure S4 Characteristic current vs. square root of scan rate recorded for NC7000, carpet, (6,5) CNTs and (7,6) CNTs.

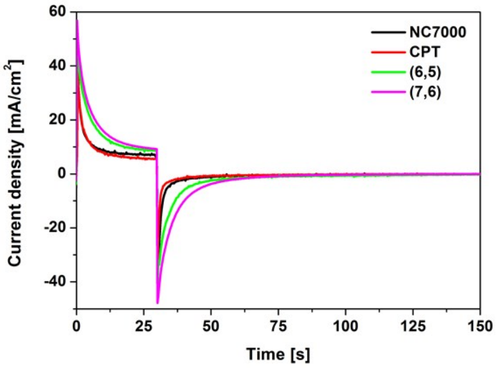

Figure S5 Chronoamperometric curves showing the potentiostatic process of charging and discharging of all experimental CNT film formulations.
